# Zeolite-Encaged Luminescent Silver Nanoclusters

**DOI:** 10.3390/ma16103736

**Published:** 2023-05-15

**Authors:** Ling Pan, Song Ye, Xinling Xv, Peixuan Lin, Ruihao Huang, Deping Wang

**Affiliations:** School of Materials Science and Engineering, Tongji University, Shanghai 201804, China

**Keywords:** zeolite, silver nanoclusters, luminescence properties, spectral manipulation, luminescence mechanism, applications

## Abstract

Silver nanoclusters (Ag NCs) are nanoscale aggregates that possess molecular-like discrete energy levels, resulting in electronic configuration-dependent tunable luminescence spanning the entire visible range. Benefiting from the efficient ion exchange capacity, nanometer dimensional cages, and high thermal and chemical stabilities, zeolites have been employed as desirable inorganic matrices to disperse and stabilize Ag NCs. This paper reviewed the recent research progresses on the luminescence properties, spectral manipulation, as well as the theoretical modelling of electronic structure and optical transition of Ag NCs confined inside various zeolites with different topology structures. Furthermore, potential applications of the zeolite-encaged luminescent Ag NCs in lighting, gas monitoring and sensing were presented. This review concludes with a brief comment on the possible future directions in the study of zeolite-encaged luminescent Ag NCs.

## 1. Introduction

Noble metal nanoclusters are new types of ultra-small luminescent centers that have different electronic structures compared with their bulk counterparts and exhibit size-related luminescence properties as quantum dots. Ag NCs are one of the most researched high luminescent metal nanoclusters, which can be effectively excited by UV light, and the resulting emission is tunable throughout the visible region with a quantum yield approaching that of commercial phosphors. The extraordinary luminescence properties of Ag NCs have attracted considerable research interest in many applications, such as photocatalysis, imaging, illumination, detection and biosensors [1,2,3,4,5]. The emissive character of Ag NCs is closely related to their constitution, geometric structure and chemical state. So far, the explicitly reported luminescent Ag NCs consist of only a few Ag^+^/Ag^0^, for example, the Ag_2_^+^ dimer [6], the Ag_3_^n+^ trimer [7], the Ag_4_^2+^ tetramer [8], and the Ag_6_^2+^ hexamer [9]. 

Due to the high surface energy, Ag NCs are very easily agglomerated into larger silver nanoparticles (Ag NPs) with surface plasma resonance (SPR) absorption, resulting in the quenching of radiative emission. In order to obtain bright and tunable emission, suitable scaffolds are required to stabilize the isolated metal nanoclusters by limiting their overgrowth and, meanwhile, offer proper crystal fields and site symmetry [10,11,12]. The incorporations of Ag NCs with organic molecules, such as DNA/RNA, proteins, and polymers, result in satisfied biocompatibility, brightness, and photostability, making them applicable as fluorescent biomarkers; however, the loss of stability over time may limit their applications in devices [13,14,15,16]. Inorganic scaffolds for Ag NCs include glasses [17,18,19,20], metal-organic frameworks (MOFs) [21,22,23], and zeolites [7,8,24]. The rigid structure of glasses can limit the aggregation of Ag NCs due to immobility, but it is harder to limit their growth precisely. The weaker thermal and chemical stability of MOFs compared with inorganics and the introduction of organic templates may hinder the control of Ag NCs growth and meanwhile bring the risk of environmental contamination.

Zeolites are desirable scaffolds for the stabilization of Ag NCs due to their efficient ion exchange capacity that allows the efficient intake of Ag^+^ and molecular-sized cages for the confinement of domain-limited functional Ag NCs. The basic units of the zeolite framework are silicon-oxygen tetrahedra [SiO_4_]^4−^ and aluminum-oxygen tetrahedra [AlO_4_]^5−^. Due to the presence of [AlO_4_]^5−^, the zeolite framework is negatively charged, and therefore some extra framework cations, such as Na^+^, K^+^, Li^+^, Ca^2+^, are usually introduced for charge balance [25]. The luminescence properties of Ag NCs are not only sensitive to the topology of the zeolite but can also be well manipulated by adjusting the Si/Al ratio and the extra framework cations of the host zeolite, the silver loading degree, as well as the external energy activation condition. Therefore, the diverse topology structures and multiple modification methods of zeolites enable easier and more flexible control of the luminescence properties of Ag NCs. Additionally, the hydration state and adsorption of noxious fume also influence the luminescence performance of Ag NCs that are confined in some types of zeolites or formatted under mild external energy activation, indicating a new sensor and detector application vista besides lighting and display. 

This short review started with the preparation and structural characterization of various zeolites-encaged luminescent Ag NCs, followed by the recent studies on the luminescence properties, spectral manipulation, luminescence mechanism and quantum chemistry-based computational study of Ag NCs in zeolites. The applications of zeolite-encaged Ag NCs in white LEDs, tunable luminescence, and moisture and gas monitoring were also presented. This review is finalized by a short comment on the particularly interesting area of future research, including the spectral modification methods, the understanding of the luminescence mechanism, and the possible applications of the high bright zeolite-encaged Ag NCs. 

## 2. Radiative Emission and Spectral Manipulation of Ag NCs in Zeolites

### 2.1. Building and Characterization of Zeolite-Encaged Ag NCs

Research on the luminescent Ag NCs-loaded zeolites started decades ago when researchers first discovered the radiative emission of Ag NCs in LTA and FAU zeolites under UV excitation [26,27,28]. To obtain a bright emission of Ag NCs with zeolites as scaffolds, a low Si/Al ratio of the host zeolite is essential as it is beneficial for the efficient intake of Ag^+^ through the replacement of extra framework cations, such as Li^+^, Na^+^, and NH^4+^, during liquid ion-exchange process. Subsequently, the application of external energies, such as thermal treatment [29,30], electron beam [31], and X-rays irradiations [32,33], may promote the reduction of Ag^+^ into Ag^0^ by trapping electrons from the O^2−^ that belonged to zeolite framework or adsorbed water, and induce the aggregation of Ag^+^ and Ag^0^ for the formation of Ag NCs in zeolites. Recent research indicated that Ag^+^ exchange efficiency and the external energy driving obviously influence the luminescence behavior of Ag NCs, as it controls the formation efficiency and distribution of Ag NCs inside zeolites [34].

**Figure 1:**
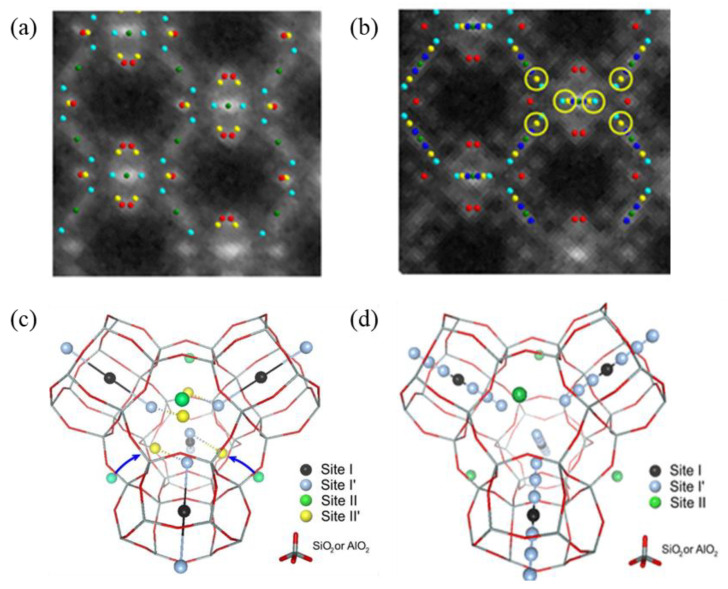
Overlay between the averaged HAADF-STEM images and the silver atomic positions obtained from XRD along the [110] zone axis for Ag-FAUX (**a**) and FAUY (**b**) zeolites in luminescent states, and three-dimensional structural models for Ag-FAUX (**c**) and FAUY (**d**) [36].

Benefiting from the advanced characterization techniques, the zeolite-encaged Ag NCs can now be observed directly [35,36,37,38]. One example is the study on the correlation between the atomic-scale structure and the luminescence properties of Ag NCs in FAU zeolite, in which complete three-dimensional structural characterizations of Ag NCs were performed by the combination of XRD Rietveld analysis and high-angle annular dark field scanning transmission electron microscopy (HAADF-STEM), as shown in Figure 1a,b. In this study, two different luminescent clusters were identified after thermal treatment, one is the [Ag_4_] cluster in Ag-FAUX zeolite, and the other one is the [Ag_3_] cluster in Ag-FAUY zeolite. It was reported that the [Ag_4_] clusters associated with yellow emission are constituted by the Ag^+^/Ag^0^ that are mainly populated at sites I, I’, II and II’, as shown in Figure 1c. The linear [Ag_3_] clusters associated with green emission are located along the hexagonal prism connectors and appear to interact weakly with Ag^0^ located at sites I, I’ and II, as shown in Figure 1d. This work laid the foundation for the luminescence mechanism investigations in terms of structure [36].

The electron spin resonance (ESR) characterization provided structural information of the confined Ag NCs [7,39,40,41]. According to the ESR analysis, the 690 nm emission in LTA zeolites is associated with Ag_6_^+^ with a doublet electronic ground state, while the 550 nm emitter is related to Ag_3_^+^ [40]. Additionally, the more powerful extended X-ray absorption fine structure (EXAFS) and the X-ray excited optical luminescence-EXAFS (XEOL-EXAFS) was also employed to obtain the direct structural information of Ag NCs formatted inside LTA, FAUY, and FAUX zeolites, such as Ag-Ag, Ag-O, and Ag-Li distances [9,42,43,44]. In recent years, a series of Ag NCs models have been built by taking the sodalite cavity of LTA zeolite as framework [8,45,46], aimed to calculate the energy level structure and possible optical transitions between the discrete energy levels of Ag NCs with different structural configurations and valence states. The combination of experimental characterization and theoretical calculation is helpful for the systematical investigation on the luminescence mechanism of Ag NCs confined inside zeolites with different topologies from the perspectives of constitution, configuration, energy level structure, and luminescence kinetics, providing a strong support for achieving bright, efficient, and tunable emission of zeolite-encaged Ag NCs.

### 2.2. Luminescence Properties of Ag NCs Confined Inside Zeolites

Low Si/Al ratio zeolites, such as FAU, LTA and SOD, have been widely employed as ideal scaffolds for Ag NCs. These zeolites all consist of sodalite cages, the differences in structure are that the sodalite cages are connected by a double six-membered ring (D6r) in FAU, a double four-membered ring (D4r) in LTA, while SOD consists entirely of the sodalite cages with a homogeneous structure [47]. Recent studies indicated that the luminescence quantum yields of Ag NCs are strongly dependent on the topology structure of the host zeolites, which can reach around 97% in FAU zeolite while below 83% in LTA zeolite [48,49]. 

FAU zeolites can be subdivided into FAUX (1 < Si/Al < 1.5) and FAUY (1.5 < Si/Al < 3) types according to the Si/Al ratio. Generally, the Ag NCs confined inside FAUY zeolite exhibit a blue-shifted emission compared with those confined inside FAUX zeolite due to the reduced lattice parameter. It was reported that the strong green visible emission centered at 540–550 nm and around 530 nm can be obtained after thermal treatment of the Ag^+^-exchanged FAUX and FAUY zeolites, respectively [24,43,50,51]. According to emission spectra, XPS studies and extended Hückel molecular orbital calculation, the optimum geometry of Ag_3_^n+^ is nearly linearly coordinated inside the D6r cage of FAU zeolites [7,50]. 

The correlation between the radiative emission and the structural configuration of Ag NCs confined inside LTA zeolites has been investigated with the help of ESR and EXAFS measurements. It was reported that the Ag NCs formed in LTA zeolites tend to be located in the sodalite cages, in which the Ag_5_^3+^ cluster formed in Ag_12_-LTA zeolite exhibits a yellow emission around 577 nm [52], the Ag_6_^+^ cluster gives off a red emission [35,41], the Ag_3_^n+^ and Ag_4_^n+^ clusters show a yellow-green emission around 550 nm [7,28,40,41], respectively. The effect of the silver loading degree on the luminescence properties of Ag NCs was also extensively explored in Li-LTA zeolites, and it was indicated that the emission wavelength of Ag NCs under UV excitation is strongly dependent on silver loading degree: Ag_1–2_Li-LTA zeolite emits green light, Ag_3–4_Li-LTA zeolite emits yellow light, and Ag_5–12_Li-LTA zeolite emits red light, respectively [34]. 

The tunable emission of Ag NCs was also reported in SOD zeolites. For example, by substituting the extra framework cations of Na^+^ with Cs^+^ and adjusting silver loading degree, the emission color of Ag NCs can be turned from green through yellow to red. The reported tunable emission of Ag NCs is mainly due to the lattice shrinkage or expansion of the host zeolites, which conversely applies the structural feedback to the luminescence property of Ag NCs [25,53,54].

### 2.3. Luminescence Tailoring through Structure Modification of Host Zeolites

**Figure 2:**
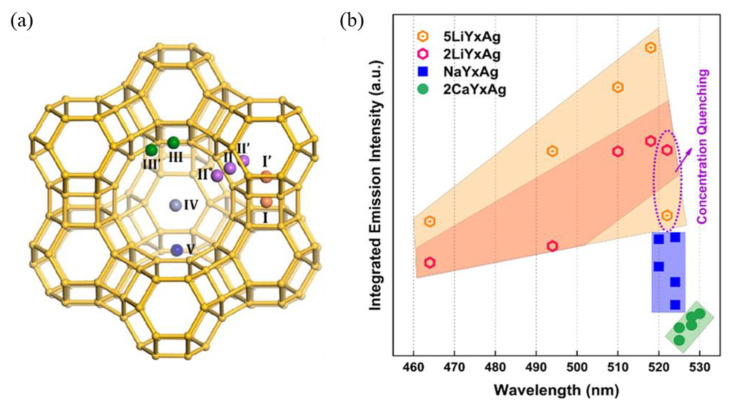
Typical extra framework cation sites I(I’), II(II’, II*), III(III’), IV, and V in the structure of FAU zeolites (**a**) [57], and summary of the integrated emission intensity and peak position of MYxAg (M = 5Li, 2Li, Na, and 2Ca; x = 5, 10, 20, 30, and 50) (**b**) [29].

Many recent studies revealed that the extra framework cations influence not only the Ag^+^ exchange capacity but also the formation efficiency and the local crystal fields of Ag NCs. Generally, small radius extra framework cations lead to reduced lattice parameters and, therefore, blue-shifted emission, and the Ag NCs showed the strongest emission intensity in Li-zeolites among a series of M-zeolites (M = Na^+^, K^+^, Cs^+^, Ca^2+^) [29,53,55,56]. Taking FAU zeolite as an example, as shown in Figure 2a, site I is located inside the D6r cage, sites II and III inside the sodalite cage, and sites IV and V inside the super cage, respectively. All the sites from I to V can be occupied by extra framework cations with different ionic radii, such as Li^+^, Na^+^, K^+^, NH^4+^, H^+^, and Ca^2+^ [55,57]. The researchers studied the influence of extra framework cations on the luminescence properties of Ag NCs in the Na-FAUY zeolite and Li^+^, Ca^2+^ partially exchanged zeolites. It was suggested that Ag NCs show stronger emission in Li-FAUY zeolites, and meanwhile, the emission peak is tunable between 462 and 530 nm, which drastically blue-shifted in Li-FAUY zeolites while slightly red-shifted in Ca-FAUY zeolites compared with in Na-FAUY zeolites, as shown in Figure 2b [29]. It was also reported that the peak emission wavelength of Ag NCs can also be well turned from 470 to 610 nm when confined in the Li^+^, Na^+^, K^+^, Cs^+^, and Mg^2+^ partially exchanged SOD zeolites [25,53,54].

**Figure 3:**
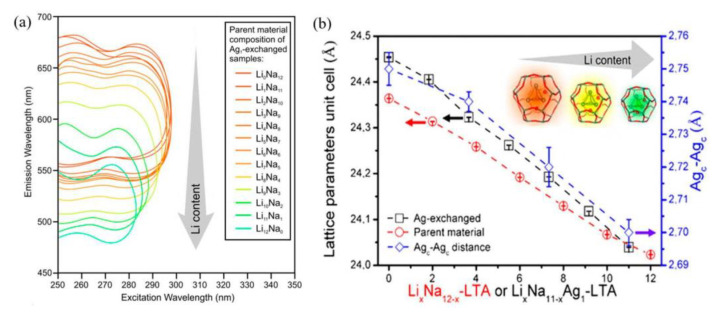
Two-dimensional excited emission spectra of Ag-LTA zeolites with various concentrations of Li^+^ exchange (**a**), variations in lattice parameter of unit cell of Li_x_Na_12−x_-LTA and Li_x_Na_11−x_Ag_1_−LTA zeolites and Ag_c_−Ag_c_ distances of the tetrahedral Ag NCs in Li_x_Na_11−x_Ag_1_-LTA zeolites (**b**) [44].

Furthermore, the effect of extra framework cations on the luminescence performance of Ag NCs was systematically studied with LTA as host zeolite by increasing the substitution ratio of Na^+^ by Li^+^. According to XEOL-EXAFS and Transmission-EXAFS (Tr-EXAFS) measurements, the Ag_c_-Ag_c_ equilibrium distance in the ground state is about 2.68 Å, while the Ag_c_-Ag_c_ equilibrium distance in the excited state is Li^+^ content dependent, which decreases from 2.75 Å in Ag_1_-exchanged Li_0_Na_12_-LTA to 2.70 Å in Ag_1_-exchanged Li_12_Na_0_-LTA. The decreased difference in Ag_c_-Ag_c_ distances between the excited state and ground state of Ag NCs from 0.07 to 0.02 Å revealed a stronger guest–host–guest interaction in the LTA zeolites with increasing Li^+^ as extra framework cations. It was indicated that the emission peak of Ag NCs shows a drastic blue shift from 605 to 510 nm with increasing Li^+^ exchange amount in Li_x_Na_12−x_-LTA zeolites because of the reduced lattice parameters of the host zeolites, as shown in Figure 3 [44].

**Figure 4:**
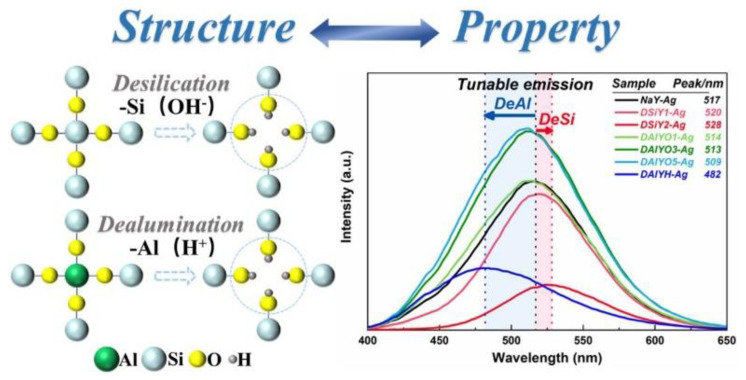
Schematic description of desilication and dealumination processes, and the emission spectra of NaY−Ag, DSiY1−Ag, DSiY2−Ag, DAlYO1−Ag, DAlYO3−Ag, DAlYO5−Ag, and DAlYH−Ag [58].

In addition to the extra framework cations substitution, the luminescence properties of Ag NCs can also be manipulated through framework modification by using desilication and dealumination approaches. During the desilication and dealumination processes, the Si/Al ratio of the zeolite framework can be modified, thus introducing a new method for regulating and optimizing the luminescence performance of Ag NCs in zeolites [58,59,60]. It was presented that the Ag NCs showed red-shifted emission in the desilicated FAUY zeolites and blue-shifted emission in the dealuminated FAUY zeolites, and a tunable emission in the wavelength range of 482–528 nm was obtained, as shown in Figure 4. This tunable luminescence property of Ag NCs is the result of controlling the local crystal field and the coupling between the host lattice and the luminescent center. Compared with adjusting the silver loading degree, the framework modification can tailor the emission of Ag NCs in a wider wavelength region and improve luminescence intensity [58].

### 2.4. Water Manipulated Ag NCs Emission

The three-dimensional eight-ring diffusion channels present in LTA zeolites allow for rapid adsorption/desorption of H_2_O; therefore, the luminescence of Ag NCs confined inside LTA zeolites is closely associated with hydration and dehydration states of the host zeolites [61,62]. Related studies suggested that the reversible luminescence response in the hydration and dehydration cycle is related to the structural dynamics of Ag NCs confined in the sodalite cages of LTA zeolites [9,34,63,64].

In the hydrated states, the Ag_4_ cluster [Ag_4_(H_2_O)_x_, 0 ≤ x ≤ 8] is responsible for the green-yellowish emission in the range of 520–570 nm under UV excitation. According to the ESR and EXAFS investigations, the luminescent [Ag_4_(H_2_O)_4_]^2+^ clusters are distributed along the six-ring axis of the sodalite cage [34,63,64]. Upon dehydration, [Ag_4_(H_2_O)_x_] clusters are transformed into the non-luminescent [Ag_6_(O_F_)_14_]^2+^ clusters that appear to interact strongly with zeolite framework oxygen (O_F_) [9]. This reversible Ag_4_^2+^↔Ag_6_^2+^ switching highlights the importance of the Ag NCs charge and valence electrons in the modulation of their optical properties.

**Figure 5:**
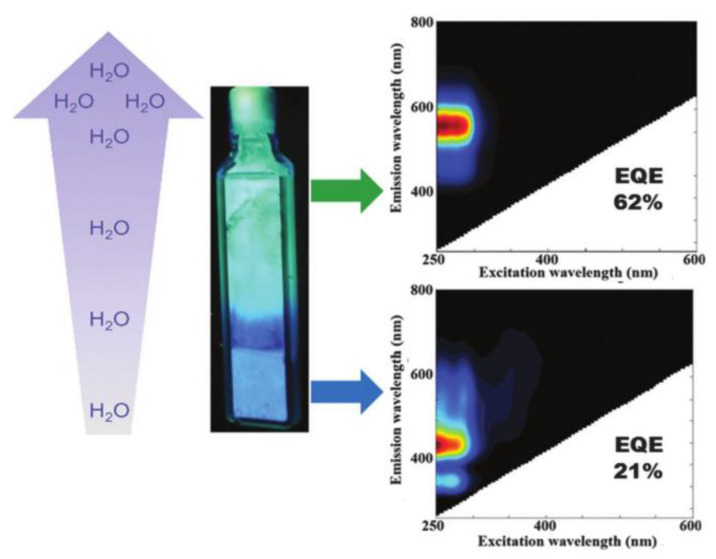
Water dependence of the emission color observed in Ag_1_Li-LTA zeolite. In fully hydrated samples (19% water content) a green emission was found, whereas in partially hydrated samples a (2% water content) blue emission was recorded [34].

The effect of hydration degree on the luminescence properties of Ag NCs has also been extensively investigated at low silver loading degrees. As shown in Figure 5, the emission of Ag_1_Li–LTA zeolites shows red-shifted emission from blue to green-yellow with increasing hydration levels [34]. This hydration/dehydration-induced emission wavelength change of Ag NCs can also be observed when confined inside K-, Na- and Ca-LTA zeolites; however, the response is not as fast as in Li-LTA zeolite, which may be due to the lower mobility of those extra framework cations than Li^+^ [9]. It was also noted that the external quantum efficiency (EQE) of Ag NCs in K-, Na- and Ca-LTA zeolites is below 16%, while Ag_1_Li-LTA zeolite has a high EQE of up to 62%, due to the pronounced dynamic change in the emissive color of the Ag_1_Li-LTA zeolite with the degree of hydration and the high EQE, which can be potentially applied as a humidity sensor.

### 2.5. Emission of Ag NCs in Rare Earth Ions Exchanged Zeolites

**Figure 6:**
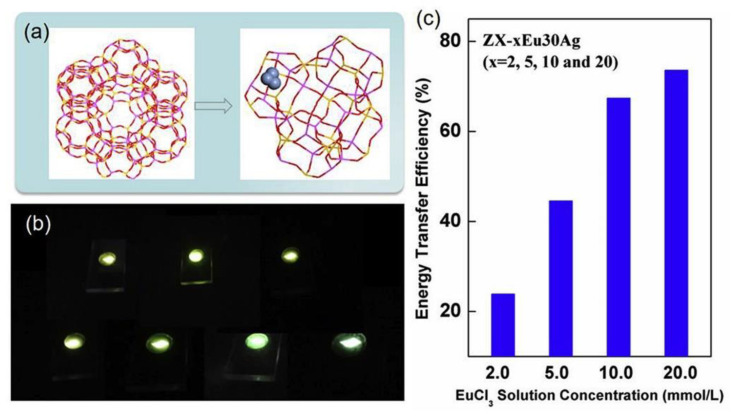
Three-dimensional structure of Na-FAUX zeolite viewed along the [111] direction and Ag NCs located in the D6r cage (**a**), physical diagram of samples with different ratios of Ag^+^/Eu^3+^ exchange concentration (**b**) and energy transfer efficiency from Ag NCs to Eu^3+^ (**c**) [51].

In recent years, the rare earth (RE) ions and Ag NCs co-loaded zeolites have been developed for the generation of a bright visible emission, in which the Ag NCs act as the sensitizer for RE ions and the RE ions regulate the formation efficiency and luminescence properties of Ag NCs [50,65]. In the Ag^+^-RE ions dual exchanged zeolites, Eu^3+^ may promote the formation of Ag NCs in Na-FAUX zeolites during the thermal treatment process, which benefits the reduction of Ag^+^ [65], while the existence of Tb^3+^ can hinder the supply of electrons required for the reduction of Ag^+^ to Ag^0^ and thus hinder the effective formation of Ag NCs [50]. Moreover, the XPS measurement indicated that the prior Tb^3+^ exchange might lead to a reduced Si/Al ratio and thus result in blue-shifted emission and stronger ionic properties of Ag NCs. Due to the tunable broadband emission that can fully overlap the excitation band of RE ions, Ag NCs are expected as effective sensitizers for RE ions in zeolites. It was reported that the energy transfer efficiency from Ag NCs to Eu^3+^ in FAUX reaches around 73% with optimized Ag^+^ exchange amount and thermal treatment condition, as shown in Figure 6 [51]. Benefiting from the efficient energy transfer from [Ag_2_]^n+^ pairs to Eu^3+^ and the combination of the red emission of Eu^3+^ and the green emission of Ag NCs, the warm white light emission was obtained in SOD zeolite [66].

## 3. Studies on the Luminescence Mechanism of Ag NCs in Zeolites

**Figure 7:**
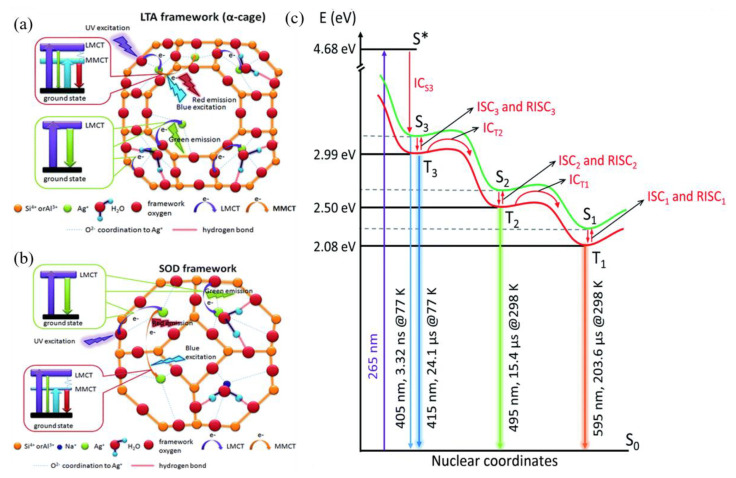
Proposed emission mechanisms in Ag^+^ exchanged LTA (**a**) and SOD (**b**) zeolites [54]; simplified kinetic electron relaxation scheme for the processes observed upon photoexcitation of Ag_1_Li_6_Na_5_−LTA zeolite (λ_ex_ = 265 nm), S* is Franck–Condon excited state (**c**) [68].

The mechanism behind the tunable luminescence of Ag NCs in zeolites attracts great research interest and has been investigated from the aspects of experimental characterization and theoretical calculation. As early as 1962, researchers discovered that Ag NCs loaded Na-LTA zeolites are colorless in a hydrated state, while activating to yellow when highly vacuumed at room temperature and turning brownish red when heated [26]. This hydration state dependence of reversible color change was attributed to electronic charge transfer from the oxygen lone pairs of the zeolite framework to the empty 5 s orbital of Ag^+^ ions, and known as the ligand-to-metal charge transfer (LMCT) process [67]. Recent studies suggested that the electron transition from the neighboring Ag^+^-Ag^+^ metal-to-metal charge transfer (MMCT) state to the ground state may be responsible for the green- and red-dominated emissions in Ag NCs loaded LTA zeolites, as shown in Figure 7a. A similar luminescence mechanism was also proposed for the Ag NCs that are confined inside SOD zeolites, as shown in Figure 7b [54]. The mechanism of Ag NCs in Li^+^ partially exchanged Na-LTA zeolites was also investigated with the help of temperature-dependent luminescence. It was suggested that the emissions located around 595 (298–233 K), 495 (233–153 K), and 395 nm (153–83 K) are due to the radiative transitions from the triplet states to the singlet states that resulted from the confinement effect of Ag NCs, and the difference between the lowest multiple minima in the excited state potential energy surface lead to different emissions, as shown in Figure 7c [68].

**Figure 8:**
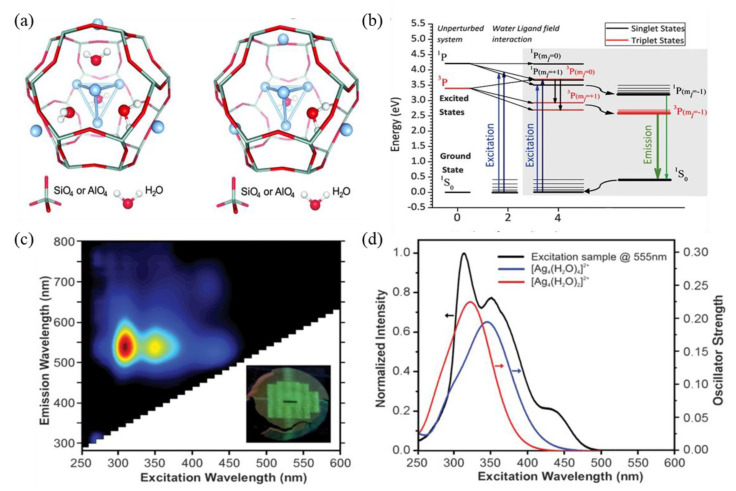
Distribution of [Ag_4_(H_2_O)_2_]^2+^ and [Ag_4_(H_2_O)_4_]^2+^ in LTA zeolite sodalite cage (**a**), energy level structure diagram (**b**), two–dimensional excitation–emission plot, inset: the picture of an X–ray–irradiated sample under 366 nm illumination (**c**), and excitation spectrum of Ag_3_K_9_−LTA zeolite (λ_em_ = 555 nm) and modeled absorption spectra for the [Ag_4_(H_2_O)_2_]^2+^ and [Ag_4_(H_2_O)_4_]^2+^ isomers (**d**) [8].

In order to acquire more information on the relationship between the structural configuration and the luminescence performance of Ag NCs, the DFT and TD-DFT calculations were carried out to compute the energy level structure, the HOMO and LUMO states, and the absorption spectra. In these computational studies, the unit sodalite cavity of [Si_24_H_24_O_36_] in LTA zeolite was used to encage Ag NCs [8,45,46,69]. One of the successful examples is the investigation of the origin of the bright-green emission of Ag NCs confined inside LTA zeolites. According to XEOL, EXAFS, TD-DFT calculation and time-resolved spectroscopy, a mixture of tetrahedral [Ag_4_(H_2_O)_x_]^2+^ (x = 2 and 4) clusters occupies the center of a fraction of the sodalite cages, as shown in Figure 8a, and the radiative emission is originated from a confined two-electron superatom quantum system with hybridized Ag and water O orbitals delocalized over the cluster. Based on the calculated energy level in Figure 8b, the modeled absorption spectra peaked at 343 and 320 nm for [Ag_4_(H_2_O)_2_]^2+^, and [Ag_4_(H_2_O)_4_]^2+^ isomers are in excellent agreement with the experimentally measured absorption and excitation spectra, as shown in Figure 8c,d. It was estimated that the Ag_3_K_9_-LTA zeolite consists of about 40% of Ag_4_(H_2_O)_4_ and 60% of Ag_4_(H_2_O)_2_ clusters [8]. 

In recent research, the all Si zeolite model was further improved by taking framework Al into consideration; the researchers designed a sodalite cavity with different Si/Al ratios of 11, 5 and 1, respectively [69]. TD-DFT calculations show that the maximum positions of calculated absorption spectra are between 3 and 4 eV, which is consistent with the experimentally observed absorption peak positions. When the framework Si is replaced by Al, the triplet state energy is increased, and this asymmetric local structure is more favorable to the intersystem crossing of different excited spin states.

## 4. Applications of Zeolite-Encaged Ag NCs

The Ag NCs show bright and tunable emission when confined inside different zeolites, and their luminescence stability is host zeolite and preparation condition dependent. For the zeolite-encaged Ag NCs with stable and tunable emission, their potential application as LED phosphors has been demonstrated, while for those exhibiting sensitive luminescence properties to environmental variations, they can be developed as sensors for toxic and hazardous gases.

### 4.1. Phosphors for LEDs

White LEDs are replacing conventional lighting devices due to the advantages of being efficient, mercury-free and environmentally friendly. Recent research indicated that the LEDs fabricated with light-emitting metal-cluster-loaded zeolites provide not only light quality but also high stability and long life [70,71]. 

**Figure 9:**
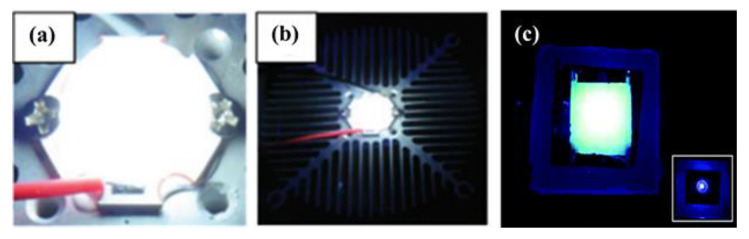
White-emitting block on a COB device driven by current (**a**,**b**) [6], remote phosphor LED prototype using a thin layer of the green luminescent dehydrated Ag_1_Li_11_–LTA zeolite (**c**) [48].

It was reported that the high thermal treatment temperature of 950 °C led to the structure collapses of Ag-loaded FAUY zeolite, and the formatted [Ag_2_]^+^ and [Ag^+^]_2_ clusters in the glassy host give off intense white light emission under UV excitation. Combining this white emitting phosphor with a UV-COB, the white LED with a CCT of 5986 K and a high Ra of 92.3 was successfully fabricated, as shown in Figure 9a,b [6]. The Ag NCs confined in Li-LTA zeolite exhibit bright green emission under 340 nm excitation, based on which the prototype green-emissive LED was fabricated using a 340 nm chip, as shown in Figure 9c, which shows uniform green emission from the phosphor layer with an EQE of 83% [48]. Alternatively, the introduction of luminescent Ag NCs-loaded LTA zeolites as emitters in a conductive polymer matrix has led to the emergence of a new type of LEDs named ZEOLEDs. By adjusting the initial silver-loading degree in zeolites, it is possible to achieve tunable emission from blue to red and even near-white [72].

### 4.2. Detection and Sensor

**Figure 10:**
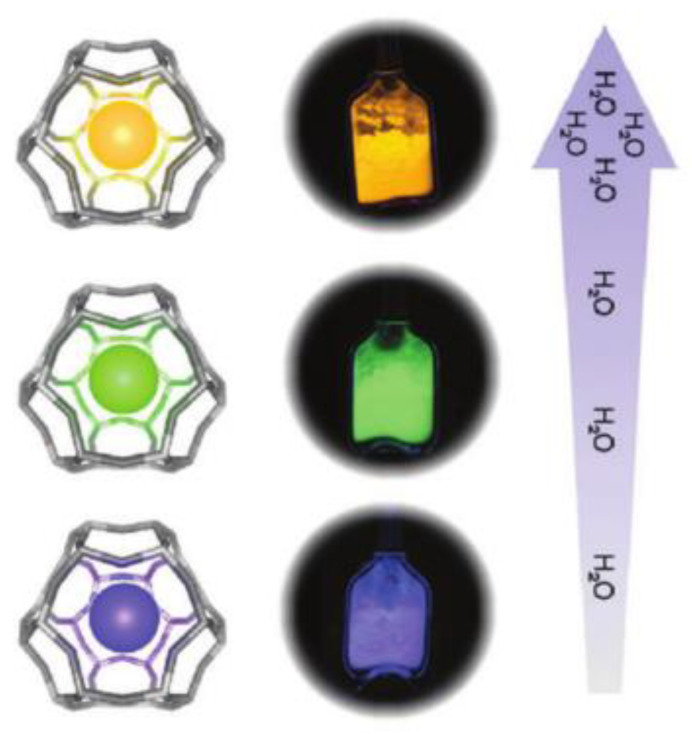
A scheme displaying the color change of real samples (Ag_1_Li–LTA zeolite) with respect to the water content is depicted in the low-right panel [34].

The optical response of zeolite-encaged Ag NCs to external stimuli can also be applied to monitoring and sensing. For example, the reversible luminescence of Ag NCs in LTA zeolites is closely related to the hydration–dehydration process, providing new ideas for humidity sensors [9,34,63,73]. Recent research indicated that the emission wavelength of Ag NCs formatted in low silver-loaded Li-LTA zeolite is significantly related to the hydration state. The fully hydrated Ag_1_Li-LTA composites (19% water content) show yellow emission, the partially hydrated samples (2% water content) show green and blue emission, and samples with less than 1% water content show blue emission, respectively, as demonstrated in Figure 10 [34]. It was suggested that this luminescent LTA(Li)-Ag composite with dramatic hydration-state-dependent color change could be used as a moisture sensor.

**Figure 11:**
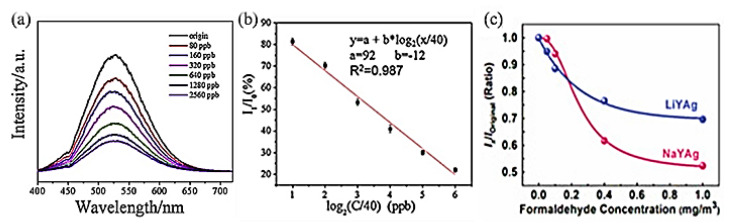
Emission spectra of Ag−FAUY under different formaldehyde concentration (**a**), and emission intensity of Ag−FAUY at different formaldehyde concentration (I_0_: original, I_1_:at different formaldehyde concentration) (**b**) [76], and relative emission intensity of Ag NCs in Li−FAUY and Na−FAUY zeolites (**c**) [77].

Formaldehyde is the main indoor pollutant emitted by buildings and decorative materials and is harmful to human health [74,75]. Recent studies indicated that the luminescence properties of FAUY zeolite-encaged Ag NCs show a rapid and sensitive response to formaldehyde gas [76,77]. Figure 11a shows the PL spectra of Ag NCs-loaded FAUY zeolites in normal and increasing concentrations of formaldehyde atmospheres from 80 to 2560 ppb. A good linear relationship between the emission intensity of Ag NCs and formaldehyde concentration in the logarithmic range was obtained, as shown in Figure 11b [76]. 

Further research indicated that the Ag NCs show different spectra response sensitivity in Na- and Li-FAU zeolites. Figure 11c shows relationships between formaldehyde concentration (x) and the emission intensity variation (y = I_x_/I_original_) of Ag CLs in Li-FAUY and Na-FAUY zeolites, which can be linearly fitted with Equations (1) and (2), respectively.

(1) $$y=A_{1}\times e^{\frac{-x}{t_{0}}}+A_{2}\cdot \;(A_{1}=0.30,\;A_{2}=0.69,\;t_{0}=0.25,\;R_{2}=0.991)$$

(2) $$y=\frac{B_{2}-B_{1}}{\left[1+{\left(\frac{x}{x_{0}}\right)}^{p}\right]}+B_{2}\cdot \;(B_{1}=1.00,\;B_{2}=0.51,\;x_{0}=0.25,\;p=2.43,\;R_{2}=0.998)$$

As can be observed from Figure 11c, AgLi-FAUY zeolite can more sensitively detect low-content formaldehyde than AgNa-FAUY zeolite, which is due to the high number of Ag^+^/Ag^0^ redox pairs in AgLi-FAUY zeolite that can promote the adsorption of formaldehyde. At higher formaldehyde concentrations, the significantly decreased emission intensity of Ag NCs in Na-FAUY zeolite is due to its relatively large specific surface area, which enables the adsorption of more formaldehyde gas. The detection limits of formaldehyde by Na- and Li-FAUY zeolite-encaged Ag NCs meet the requirements of WHO, OSHA and China’s standards for indoor air quality.

## 5. Conclusions

Zeolites are promising scaffolds for the assembly of highly luminescent Ag NCs, as they allow for the flexible regulation of the luminescence properties of confined Ag NCs due to their abundant topology structures and exchangeable extra-framework cations. So far, the tunable emission of Ag NCs nearly across the visible region has been achieved by controlling the local crystal field and guest–host–guest interaction through host zeolite structure modification. Nevertheless, there is still a necessity to explore further methods to more precisely control the emission wavelength and improve the emission intensity of Ag NCs, for example, introducing non-luminescent RE ions as extra-framework cations or making use of intergrowth zeolites as scaffolds. On the other hand, how the geometric and electronic structure of Ag NCs influences their luminescence properties should be systematically studied with the assistance of quantum-chemistry-based computation, in which the framework Al and extra-framework cations should be taken into consideration in the building of sodalite or D6r cavities. Understanding the exact luminescent and spectral control mechanisms, as well as how the various parameters governing the luminescence tunability with the help of more advanced characterization techniques and theoretical calculations, is of great importance for the design of zeolite-encaged luminescent Ag NCs for efficient lighting phosphors and smart sensor devices applications, as well as other applications in adsorption and catalysis.
